# Impact of Additives and Packing Density on Fermentation Weight Loss, Microbial Diversity, and Fermentation Quality of Rape Straw Silage

**DOI:** 10.3390/microorganisms12101985

**Published:** 2024-09-30

**Authors:** Baozhu Yang, Na Na, Nier Wu, Lin Sun, Ziqin Li, Moge Qili, Hongyan Han, Yelin Xue

**Affiliations:** 1 State Key Laboratory of Reproductive Regulation and Breeding of Grassland Livestock, School of Life Sciences, Inner Mongolia University, Hohhot 010070, China; 15148878701@163.com; 2 Inner Mongolia Engineering Research Center of Development and Utilization of Microbial Resources in Silage, Inner Mongolia Academy of Agriculture and Animal Husbandry Science, Hohhot 010031, China; 13684752695@163.com (N.N.); 15947414810@163.com (N.W.); sunlin2013@126.com (L.S.); ziqinli88@yahoo.com (Z.L.); qilmge@163.com (M.Q.)

**Keywords:** rape straw silage, packing density, bacterial community, fermentation quality, FWL

## Abstract

To investigate the effects of the combined addition of *Lactiplantibacillus plantarum* and sucrose on the fermentation weight loss (FWL), fermentation quality, and microbial community structure of ensiled rape straw under varying packing density conditions. After harvesting, the rapeseed straw was collected, cut into 1–2 cm pieces, and sprayed with sterile water to adjust the moisture content to 60%. The straw was then divided into two groups: one treated with additives (1 × 10^5^ CFU/g fresh material of *Lactiplantibacillus plantarum* and 10 kg/t fresh material of sucrose), and the other sprayed with an equivalent amount of sterile water as the control (CK). The treated materials were thoroughly mixed and packed into silos at densities of 450, 500, and 550 kg/m^3^. FWL was recorded on days 1, 3, 6, 15, 20, and 45 of fermentation. On day 45, the samples were analyzed for fermentation quality, microbial counts, and microbial diversity. FWL increased significantly (*p* < 0.05) in both the treated (LS) and control groups during fermentation. The LS group showed higher lactic acid (LA) levels (*p* < 0.05) and lower ammonia nitrogen levels (*p* < 0.05) compared to CK. The CK group had significantly higher (*p* < 0.05) counts of Coliforms and lower bacterial counts (*p* < 0.05) than LS. The dominant genera in the silage were *Xanthomonas*, *Lactiplantibacillus plantarum*, and *Lentilactobacillus*. In the LS group, the relative abundances of *Lactiplantibacillus plantarum* and *Lentilactobacillus* ranged from 16.93% to 20.43% and 15.63% to 27.46%, respectively, with their combined abundance being higher than in CK. At a packing density of 500 kg/m^3^, the relative abundances of *Lactiplantibacillus plantarum* and *Lentilactobacillus* in the LS group were significantly higher (*p* < 0.05) than in CK. Increasing packing density and applying additives to rape straw silage effectively reduced FWL, improved fermentation quality, boosted the relative abundance of beneficial lactic acid bacteria, and decreased the presence of undesirable bacteria such as *Enterobacter* and *Bacillus*.

## 1. Introduction

Rape (*Brassica napus subsp. napus* L.) is the world’s largest oilseed crop, with extensive planting areas and a wide distribution. In the 2021–2022 season, global rape production reached 68 million tons, with China contributing 19.34% of this total [1]. Rape straw, the primary by-product after seed harvesting, is a rich biomass resource [2]. The entire rape plant holds significant feed value, with the straw containing 5.24% crude protein (CP)—a higher percentage than that found in corn and wheat straws [3]. However, due to its poor palatability, high fiber content, and susceptibility to mold, rape straw’s use as animal feed is limited [4]. Consequently, large quantities of rape straw are discarded as agricultural waste, leading to significant resource loss and environmental impact. Although studies have shown that rape straw can be repurposed as organic fertilizer [5], decorative materials [6], boards, and fuel [7], its potential as a feed source remains underutilized.

Ensiling is a traditional method of preserving forage that can be easily mechanized from harvesting to feeding. It provides palatable feed during winter or dry seasons when fresh forage is unavailable [8]. Silage is now commonly used as a crucial nutritional feed source for ruminants [9]. Under anaerobic condition, adding microbial inoculants facilitates the fermentation of water-soluble carbohydrates (WSCs) in rape straw, producing lactic acid and other organic acids. This process inhibits the growth of harmful bacteria, reduces the content of detrimental substances, and preserves the nutrients in the silage [10]. Therefore, WSCs like sucrose and lactic acid bacteria inoculants are commonly used to enhance forage preservation [11]. By modifying the bacterial community and allowing lactic acid bacteria to dominate, rapid and efficient fermentation is achieved with minimal dry matter loss [12]. Given the rich nutrient content of rape straw, ensiling offers a promising approach to enhance its utilization as a ruminant feed source.

A critical factor in the ensiling process is packing density, which affects material porosity, gas exchange, and the distribution of moisture and microbial communities within the silo. These factors directly influence the rate of fermentation and the quality of the final silage product. Proper packing density is essential for minimizing fermentation losses and inhibiting the growth of undesirable bacteria [13]. However, limited research has been conducted on how different packing densities affect the fermentation weight loss (FWL), fermentation quality, and microbial communities in rape straw silage.

This study addresses this gap by investigating the effects of varying packing densities and LAB inoculation on the fermentation dynamics, microbial community structure, and overall quality of rape straw silage. Specifically, the research aims to assess how different packing densities influence FWL, fermentation quality, and the bacterial community composition in rape straw silage, providing new insights into optimizing its use as a feed resource. The novelty of this study lies in its focus on a comprehensive evaluation of the fermentation process under different packing conditions, which has not been fully explored in the context of rape straw silage.

## 2. Materials and Methods

### 2.1. Materials and Experimental Design

The rape straw used in the study was provided by the Inner Mongolia Academy of Agricultural and Animal Husbandry Sciences. After harvesting the rapeseed, the remaining straw was collected, cut into 1–2 cm pieces, and sprayed with water to adjust the moisture content to 60%. The straw was then divided into two batches as follows: one batch was treated with *Lactiplantibacillus plantarum* and *Lentilactobacillus buchneri* (provided by Sichuan Gaofuji Biological Co., Ltd., Chengdu, China) along with sucrose (provided by Shandong Gushuo Biological Technology Co., Ltd., Jining, China) (LS), while the other batch was supplemented with an equal amount of sterile water as a control (CK). The concentration of viable lactic acid bacteria in *Lactiplantibacillus plantarum* was 1 × 10^5^ CFU/g, with an addition rate of 5 g/t, and the sucrose was added at 10 kg/t. After thorough mixing, three samples taken from both the CK and LS groups were packed into plastic-sealed jars (20 cm in diameter; 30 cm in height) at fresh weight densities of 450, 500, and 550 kg/m^3^. To ensure the accuracy and reliability of the results, each group had four replicates, resulting in a total of 24 plastic-sealed jars (6 different treatments, each with 4 replicates) placed in a laboratory for fermentation.

### 2.2. Fermentation Weight Loss (FWL) Analysis

The weights of the plastic-sealed jars were measured on days 0, 1, 3, 6, 20, and 45 of fermentation, and the FWL was calculated using the following formula:

$$\begin{array}{l} FWL=\frac{\left(\mathrm{Weight}\;\mathrm{of}\;\mathrm{sample}\;\mathrm{on}\;\mathrm{day}\;0\;-\mathrm{Weight}\;\mathrm{of}\;\mathrm{sample}\;\mathrm{on}\;\mathrm{day}\;x\right)}{\mathrm{Weight}\;\mathrm{of}\;\mathrm{sample}\;\mathrm{on}\;\mathrm{day}\;0\;}*1000 \end{array}$$

Note: x represents the number of fermentation days (day 1, 3, 6, 20, and 45).

### 2.3. Fermentation Quality Analysis

After 45 days of fermentation, the plastic-sealed jars were opened, and the contents were thoroughly mixed. Ten grams of fresh sample were taken and combined with 90 mL of sterilized water. This mixture was homogenized for 2 min using a sterile homogenizer (model JX-05, Shanghai Jingxin Industrial Development Co., Ltd., Shanghai, China) and then filtered through four layers of gauze and qualitative filter paper to remove any plant residues. The resulting filtrate was collected for further analysis. The pH value was measured using a pH meter (model PB-10, Sartorius, Göttingen, Germany) [14]. The filtrate was then passed through a 0.22 μm membrane filter, and the contents of lactic acid (LA), acetic acid (AA), propionic acid (PA), and butyric acid (BA) were determined using high-performance liquid chromatography (HPLC) equipped with a KC2811 column (set at 50 °C, flow rate of 1 mL/min, and UV detector at 210 nm) [15].

For dry matter (DM) content determination, approximately 50 g of samples were placed in a 65 °C ventilated oven (model BPG-9240 A, Shanghai Yiheng Scientific Instrument Co., Ltd., Shanghai, China) until completely dried (48 h). The dried samples were then ground using a grinder (model FS-6D, Jinan Feichi Machinery Equipment Co., Ltd., Jinan, China) for further nutritional composition analysis.

### 2.4. Microbial Community Structure Analysis

DNA sequencing was applied to analyze microbial composition due to its ability to provide a comprehensive and high-resolution profile of microbial communities, especially in complex environments like silage. This method, which targets the V3–V4 region of the bacterial 16S rDNA gene, is widely recognized for its accuracy in detecting a broad range of microbial taxa, including both culturable and nonculturable species. Compared to traditional culture-dependent techniques, which are limited by the growth conditions and may miss a significant portion of the microbial community, DNA sequencing offers an unbiased and more complete analysis of the microbial diversity present in the samples [16,17]. This method has proven particularly useful for characterizing bacterial communities involved in silage fermentation, enabling the identification of key microorganisms that influence silage quality.

DNA was extracted using an EZNA^®^ Stool DNA Kit. Primers 341F (5′-CCTACGGGNGGCWGCAG-3′) and 805R (5′-GACTACHVGGGTATCTAATCC-3′) were used for polymerase chain reaction (PCR) to amplify the V3-V4 region of the bacterial 16S rDNA gene. PCR conditions were as follows: initial denaturation at 94 °C for 30 s, followed by extension at 72 °C for 45 s, and a final extension at 72 °C for 10 min. PCR products were verified through 2% agarose gel electrophoresis. Ultrapure water was used as a negative control during DNA extraction to prevent false-positive results. The PCR products were then purified and quantified using AMPure XP beads (Beckman Coulter Genomics, Danvers, MA, USA). The synthesized amplicons were assessed using the Agilent 2100 Bioanalyzer and Illumina library quantification kits (Kapa Biosciences, Woburn, MA, USA), followed by sequencing on the Nova Seq PE250 platform.

Sequencing was performed on the Illumina Nova Seq platform according to the manufacturer’s protocol. Barcode and primer sequences were assigned and truncated from paired end reads based on unique barcodes. Paired-end reads were merged using FLASH software (v0.94), and raw reads were filtered under specific conditions to obtain high-quality clean reads. Chimeric sequences were filtered out using V search software (v2.3.4). After removing duplicates with DADA2, feature tables and feature sequences were generated. The SILVA classifier was employed to normalize feature abundances. For statistical analysis, alpha diversity indices (Chao1, observed species, Goods coverage, Shannon index, and Simpson index) were calculated using QIIME2 to evaluate species richness and diversity within each sample. Beta diversity, which assesses differences in microbial community composition between samples, was also analyzed using QIIME2. Principal coordinate analysis (PCoA) was performed to visualize beta diversity, while permutational multivariate analysis of variance (PERMANOVA) was used to assess statistically significant differences between microbial communities. The R package (version 3.5.2) was employed for additional multivariate statistical analyses and visualizations.

### 2.5. Nutritional Composition Analysis

The total nitrogen (TN) content in both fresh and ensiled feed was determined using a Kjeldtec automatic analyzer (model 8400; Foss Co., Ltd., Hillerød, Denmark) with copper as a catalyst. The TN values were then multiplied by 6.25 to calculate the crude protein (CP) concentration in the silage. The WSC content was analyzed using the anthrone sulfuric acid colorimetric method [18].

## 3. Results

### 3.1. Fermentation Weight Loss 

The overall trend of fermentation weight loss (FWL) across all silages showed an increase over time, with significant effects observed from days 3 to 45 (*p* < 0.05). At a packing density of 450 kg/m^3^, the FWL of the LS treatment was significantly lower than that of the CK group from days 1 to 45 (*p* < 0.05). At 500 kg/m^3^, significant differences between LS and CK were observed only on day 3 (*p* < 0.05). The LS550 group showed the lowest FWL on day 1 compared to LS450 and LS500 (*p* < 0.05). On days 3, 6, and 20, a clear trend emerged, with FWL decreasing as packing density increased (*p* < 0.05). By day 45, LS450 had a higher FWL than both LS500 and LS550 (*p* < 0.05). This indicates that higher packing densities led to lower fermentation losses overall, with inoculation treatment and packing density both playing a significant role in reducing FWL over time (Table 1).

### 3.2. Fermentation Quality 

At all packing densities, the LS treatment resulted in a lower pH and higher levels of LA, PA, and the LA/AA ratio compared to CK (*p* < 0.05). Specifically, LS450 had a lower PA content than CK450 (*p* < 0.05). Additionally, CK550 exhibited higher BA levels than CK450 and CK500 (*p* < 0.05). The AN content in LS was consistently lower than in CK, with CK450 showing a higher AN level than both LS450 and LS500 (*p* < 0.05). Significant interactions between packing density and inoculation treatment were observed for LA and BA levels (*p* < 0.05), underscoring the importance of both variables in determining the fermentation quality of the silage (Table 2).

### 3.3. Microbial Counts 

The total bacterial count was lower in LS treatments compared to CK at packing densities of 500 and 550 kg/m^3^ (*p* < 0.05), with LS450 showing a higher bacterial count than LS500 and LS550 (*p* < 0.05). Inoculation with lactic acid bacteria (LAB) led to a significant reduction in coliform bacteria, especially at higher packing densities (LS500 and LS550) (*p* < 0.05). Significant interactions between packing density and inoculant treatment were noted for coliform counts, highlighting the combined effect of these factors on microbial dynamics in silage (Table 3).

### 3.4. Nutritional Composition 

DM, WSC, and CP were significantly influenced by both packing density and inoculation (*p* < 0.05). The DM content in LS was consistently higher than in CK, while the DM value in CK550 was lower than in CK450 and CK500 (*p* < 0.05). The WSC content in LS was higher than in CK at packing densities of 450 and 550 kg/m^3^ (*p* < 0.05). LS550 had a higher WSC content than LS450 and LS500 (*p* < 0.05). CP content was also higher in LS treatments compared to CK, though LS550 exhibited a lower CP than LS450 and LS500 (*p* < 0.05). These results demonstrate the beneficial impact of both a higher packing density and LAB inoculation in preserving nutritional content during fermentation (Table 4).

### 3.5. Bacterial Communities 

**Figure 1:**
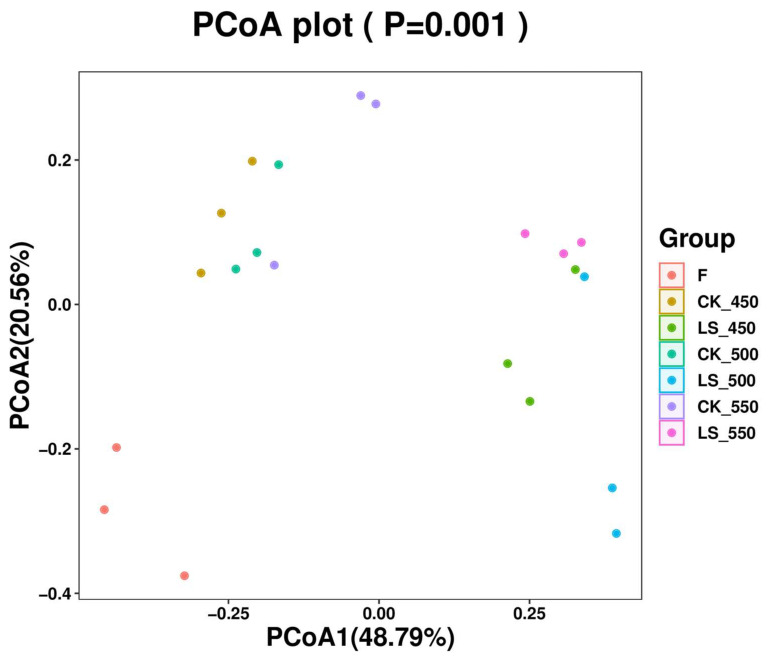
Effect of additives and packing density on principal coordinates analysis (PCoA) of bacterial community in rape straw silages (*n* = 3). Note: F, fresh material; CK, ensiled at 450, 500, and 550 kg/m^3^ (CK 450, CK 500, CK 550); LS, rape straw ensiled with LAB and sugar at the same densities (LS 450, LS 500, LS 550).

The fresh materials exhibited higher observed OTUs and indexes for Shannon, Simpson, Chao1, and Pielou_e indexes compared to all silages (*p* < 0.05). The Shannon index of LS500 was lower than that of CK500 (*p* < 0.05), while CK550 had a lower Simpson index than CK450 and CK500 (*p* < 0.05). Notably, the Pielou_e index of LS450 was higher than that of LS500 (*p* < 0.05) (Table 5). The PCoA results indicated a clear separation between the bacterial communities in fresh materials and those in all silage treatments, suggesting that the ensiling process dramatically alters the microbial community structure (Figure 1).

**Figure 2:**
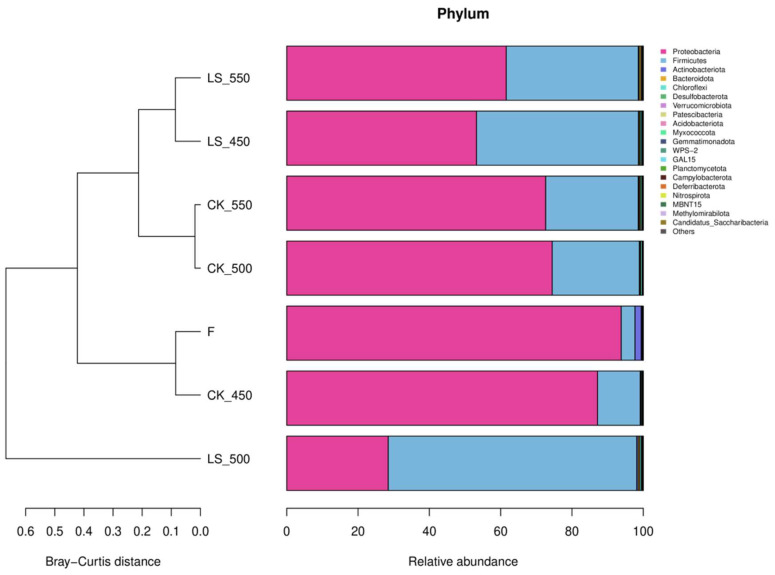
Effect of additives and packing density on relative abundance of bacterial community (phylum level) in rape straw silages (*n* = 3). Note: F, fresh material; CK, ensiled at 450, 500, and 550 kg/m^3^ (CK 450, CK 500, CK 550); LS, rape straw ensiled with LAB and sugar at the same densities (LS 450, LS 500, LS 550).

At the phylum level, Proteobacteria was dominant in the raw materials, comprising 93.81% of the community (Figure 2). After ensiling, the relative abundance decreased significantly in all treatments except CK450 (*p* < 0.05). In contrast, Firmicutes increased significantly in all groups, particularly in the LS treatments.

**Figure 3:**
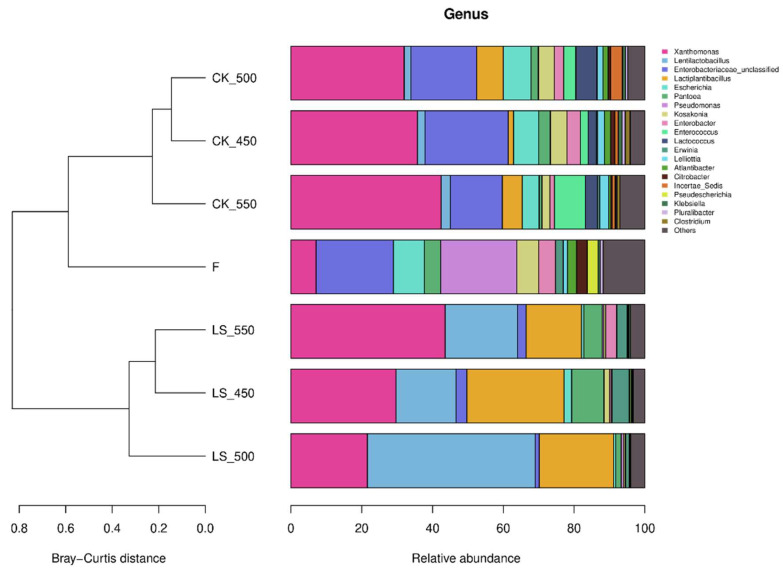
Effect of additives and packing density on relative abundance of bacterial community (genus level) in rape straw silages (*n* = 3). Note: F: fresh material; CK, ensiled at 450, 500, and 550 kg/m^3^ (CK 450, CK 500, CK 550); LS, rape straw ensiled with LAB and sugar at the same densities (LS 450, LS 500, LS 550).

**Figure 4:**
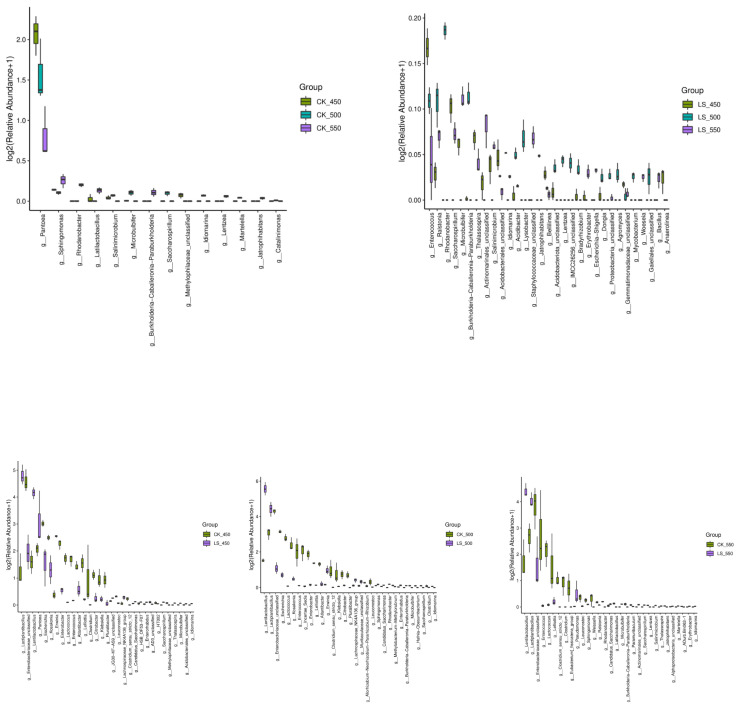
Difference in bacterial community (genus level) in rape straw silages (*n* = 3). Note: F: fresh material; CK, ensiled at 450, 500, and 550 kg/m^3^ (CK 450, CK 500, CK 550); LS, rape straw ensiled with LAB and sugar at the same densities (LS 450, LS 500, LS 550).

At the genus level, the predominant bacterial genera in the raw materials were *Enterobacteriaceae_unclassified* (21.8%), *Pseudomonas* (21.5%), *Escherichia* (8.74%), *Xanthomonas* (7.10%), *Enterobacter* (4.74%), and *Pantoea* (4.64%) (Figure 3). After ensiling, *Xanthomonas* became dominant in CK, increasing to 35.8–42.4% (*p* < 0.05). In contrast, the relative abundances of *Lentilactobacillus* and *Lactiplantibacillus plantarum* were higher in LS compared to CK. Specifically, *Lentilactobacillus* in CK450, CK500, and CK550 was 2.10%, 1.89%, and 2.63%, respectively, while in LS450, LS500, and LS550, it increased to 16.93%, 47.40%, and 20.43% (*p* < 0.05). *Lactiplantibacillus plantarum* followed a similar trend, with higher relative abundances in LS groups (27.46%, 21.01%, 15.63%) compared to CK groups (1.51%, 7.50%, 5.63%) (*p* < 0.05). Notably, LS450 had more *Lactiplantibacillus plantarum* but less *Lentilactobacillus* than LS500 and LS550 (*p* < 0.05). Spoilage-related genera such as *Enterobacteriaceae_unclassified*, *Pseudomonas*, *Escherichia*, and *Kosakonia* decreased after ensiling, while *Pantoea* was more abundant in CK450 than in CK500 and CK550 (*p* < 0.05). Additionally, *Enterococcus* was more abundant in LS450 than in LS500 and LS550 (*p* < 0.05) (Figure 4). Overall, LS had higher relative abundances of *Lactiplantibacillus plantarum* and *Lentilactobacillus* and lower abundances of *Enterobacteriaceae_unclassified* and *Escherichia* compared to CK (*p* < 0.05).

## 4. Discussion

### 4.1. Effect of Additives and Packing Density on Fermentation Weight Loss during the Fermentation Process of Rape Straw Silage

The aim of this study was to investigate the effects of additives (including lactic acid bacteria and sugar) and different packing densities on the fermentation weight loss, microbial diversity, and fermentation quality of rape straw silage. The results showed that increasing packing density and using additives could effectively reduce the weight loss and improve the fermentation quality of rape straw silage. In addition, the use of additives also increased the relative abundance of beneficial lactic acid bacteria, while reducing the presence of undesirable bacteria such as *Enterobacter* and *Bacillus*.

Anaerobic fermentation and wastewater discharge have an effect on the loss of silage during storage [19]. However, in this study, no significant effluent was observed in the plastic tanks, indicating that the primary cause of silage loss in rape straw was anaerobic fermentation during storage. During the anaerobic fermentation of silage, losses are primarily attributed to carbon dioxide production by heterofermentative lactic acid bacteria, yeast, *Enterobacter*, and *Clostridium* during storage [20,21]. In this study, *Enterobacteriaceae_unclassified* and *Escherichia* made up 21.8% and 8.7% of the fresh materials, respectively. The initial gas loss in rape straw silage could likely be attributed to the activity of *Enterobacter* and yeast.

However, in the LS treatment, the inoculation of LAB led to a rapid increase in LAB numbers at the onset of fermentation, which quickly lowered the pH by producing LA and AA. This drop in pH to below 5.0 inhibited the activities of *Enterobacteria* and yeast [20]. In this study, the addition of LAB and sugar decreased the relative abundance of *Enterobacteria* while increasing the relative abundance of LAB. As fermentation progressed, the activities of yeast and *Enterobacteria* were further suppressed, leading to a reduction in the fermentation loss rate. Over time, LAB activity became dominant, contributing to an overall increase in fermentation loss as the process continued.

The inoculation of LAB can intensify the initial fermentation of silage [14,22], resulting in a higher initial FWL [20]. This explains why the FWL in LS-treated silage was higher than in CK during the early stages of fermentation. However, the initial high fermentation intensity eventually led to reduced microbial activity [23], which in turn resulted in a lower FWL in the inoculated silage by days 20 and 45 of fermentation.

In summary, the gas loss in rape straw silage during the early stages of fermentation is likely due to the activities of LAB, *Enterobacteria*, and yeast. While the inoculation of LAB increases the initial fermentation intensity, leading to higher initial losses, this also results in reduced microbial activity and lower fermentation losses in the later stages of the process. In general, the use of additives increased the fermentation intensity during the initial stage but slowed the rate of fermentation weight loss in the later stages. A higher packing density helps reduce weight loss in the early stages, likely by limiting air penetration and reducing oxidation losses. While the overall trend of weight loss was similar across different packing densities, the absolute values differed. There is an interaction between packing density and the use of additives in influencing fermentation weight loss, with the specific effects varying depending on both the density and the type of additives used.

### 4.2. Effect of Additives and Packing Density on Fermentation Quality of Rape Straw Silage

To achieve high-quality silage, it is crucial to have a sufficient population of LA-producing bacteria to ensure a rapid decline in pH [24]. In this study, compared to CK, the pH value and ammonia nitrogen content in LS decreased, while the lactic acid content increased. These findings indicate that the addition of LAB and sugar enhances the fermentation quality of silage. Specifically, the introduction of homofermentative LAB increased the production of LA, while heterofermentative LAB promoted the formation of AA [25,26]. The *Lactiplantibacillus plantarum* used in this study likely had a positive impact early in the fermentation process by producing more LA in the inoculated silage, which led to a reduction in pH. As expected, LA levels increased in LS as the main fermentation product. Additionally, *Lentilactobacillus* may have played a key role during the later stages of fermentation, as evidenced by the higher LA/AA ratio in LS under conditions with sufficient WSCs [27,28]. The higher abundance of *Lentilactobacillus* in LS suggests it may be more active during late fermentation, converting LA into AA, which correlates with the observed increase in AA content [29]. Butyric acid in silage is undesirable because the activity of *Clostridium* can lead to secondary fermentation and cause nutritional loss. *Clostridium perfringens* is a versatile pathogen responsible for causing histotoxic infections, enteritis/enterocolitis, and enterotoxemia [30]. When it proliferates excessively in the gut, it produces potent toxins that can damage the intestinal lining, allowing intestinal contents to leak into the bloodstream. This can trigger a severe inflammatory response and tissue damage. The condition, often presenting as acute enterotoxemia, is characterized by a high fever, diarrhea, vomiting, and rapid weight loss. Without prompt treatment, enterotoxemia can be fatal. The addition of LAB and sugar significantly reduced BA content (*p* < 0.05), likely because the additives lowered the pH, thereby inhibiting *Clostridium* activity.

The lactic acid content in LS is significantly higher than in CK, indicating that the additive enhances lactic acid production, a key indicator of silage fermentation quality. The ammonia nitrogen content in LS is notably lower than in CK, suggesting that the additive reduces protein degradation, thereby preserving the nutritional value of the silage. The pH of LS is also significantly lower than CK, further confirming the additive’s positive impact on improving fermentation quality. Additionally, a higher packing density reduces fermentation losses and increases dry matter recovery, contributing to better fermentation outcomes. It also lowers ammonia nitrogen levels and minimizes protein degradation, helping to maintain the silage’s nutritional value.

### 4.3. Effect of Additives and Packing Density on Nutritional Composition of Rape Straw Silage

DM content is a critical factor influencing silage quality. Ruppel et al. noted that packing density is negatively correlated with DM loss during storage in farm-scale silos [31]. A lower DM loss in silage generally indicates better nutrient preservation [32]. In this study, the addition of LAB and sugar, along with higher packing densities, resulted in higher DM recovery rates. This may be attributed to the fact that higher packing densities limit air penetration, thereby reducing oxidation losses during silage. These findings align with Sucu et al., who reported that tightly packed silage had better DM recovery compared to loosely packed silage [33].

Ammonia nitrogen (AN) content is a key indicator of protein degradation in silage [34], mainly associated with the activity of undesirable microorganisms such as coliforms. AN represents the extent to which protein is preserved during fermentation (35). In this study, all silages showed good preservation during fermentation, with AN content decreasing in uninoculated silage as packing density increased. Previous studies have also shown that AN concentrations in uninoculated sorghum, whole-crop corn, and whole-crop barley silages decrease with higher packing densities [33,35]. This may be because, in the early stages of fermentation, a high packing density allows LAB to quickly establish an anaerobic environment through rapid fermentation in low-oxygen conditions, thereby inhibiting harmful microbial activity and reducing protein degradation by unwanted bacteria [36]. The formation of AN during the early stages of rape straw silage fermentation was likely due to the activity of *Enterobacteriaceae*, a pattern that has also been observed in barley silage [37]. In this study, the use of additives significantly reduced ammonia nitrogen content (*p* < 0.05), which corresponded with a reduction in coliform numbers in the LS treatment.

The DM and CP content in LS were significantly higher than in CK, indicating that the additive effectively reduced dry matter loss during fermentation, helping to preserve the feed’s nutritional value. It inhibited the activity of protein-degrading bacteria, thereby minimizing protein loss. Additionally, a higher packing density improved dry matter recovery, further contributing to the preservation of dry matter content in the silage.

### 4.4. Effect of Additives and Packing Density on Bacterial Communities of Rape Straw Silage

It was found that there were no significant differences in the alpha diversity index of bacterial communities between inoculated and non-inoculated silage, nor with varying packing densities. This finding is consistent with previous studies on sweet sorghum, barley, and Sorghum-Sudangrass silage, which have shown that these factors have little impact on the alpha diversity of silage bacterial communities [23,35,38]. However, the addition of LAB and sugar during ensiling did increase the Shannon, Simpson, and Pielou_e diversity indices, indicating that LAB inoculation can enhance the alpha diversity of bacterial communities in rape straw silage (*p* < 0.05) [23]. Principal coordinate analysis (PCoA) further revealed that CK and LS silage samples were grouped according to their bacterial community structure. Previous studies have also shown that bacterial communities in sweet sorghum, whole wheat silage, and Sorghum-Sudangrass silage tend to cluster together as packing density increases, indicating that silages of different densities tend to have similar bacterial communities [23,35,38]. Microbial abundance analysis revealed that Proteobacteria and Firmicutes were the dominant phyla in both the raw feedstock and all treatment groups. The dominance of these two phyla may be attributed to the low pH and anaerobic conditions of silage, which favor their growth. Firmicutes play a critical role in silage by converting sugars into lactic acid, which is essential for preserving the feed. Proteobacteria are involved in the early stages of the fermentation process and contribute to the breakdown of proteins and amino acids [39,40]. The addition of LAB and sugar increased the relative abundance of Firmicutes, suggesting that these additives can alter the bacterial community structure in silage. This finding is consistent with results reported by Yuan and Wan et al. [41,42] and may be related to the increased presence of *Lentilactobacillus* and *Lactiplantibacillus plantarum*, both of which belong to Firmicutes. In addition, *Lentilactobacillus* and *Lactiplantibacillus plantarum* were found to be the dominant bacteria during the fermentation of silage treated with additives. Most studies indicate that the microbial communities involved in lactic acid fermentation in silage are primarily composed of *Lactobacillus*, *Pedicoccus*, *Lactococcus*, *Weissella*, and *Leuconostoc* [43]. The higher proportion of LAB in these communities may be due to their dominance, which lowers the pH and inhibits undesirable microorganisms. As the number of dominant bacteria increases, microbial diversity tends to decrease, which is one of the hallmarks of successful silage fermentation [44]. The use of silage additives significantly altered the structure of the bacterial community, as also reported [45]. Typically, the complex microbial community in raw materials is gradually replaced by LAB, leading to a rapid decrease in microbial diversity, another indicator of successful fermentation [32]. Liu et al. suggested that *Pantoea* might compete with LAB for nutrients and contribute to the production of ammonia nitrogen, while the presence of *Clostridium* in silage is undesirable [46]. *Clostridium* can degrade lactic acid, leading to an increase in pH and a decrease in the nutritional value of the forage through amino acid dissimilation, resulting in fermentation losses and reduced livestock consumption [47]. Overall, the abundance of *Pantoea* and *Clostridia* was low during the ensiling process.

The abundance of *Lactobacillus plantarum* and *Lentilactobacillus* inoculated silage was higher than in CK across different packing densities, likely due to the LAB additive used and its fermentation characteristics. In CK, the relative abundance of *Lentilactobacillus* was higher than that of *Lactiplantibacillus plantarum*, but as packing density increased, the relative abundance of *Lactiplantibacillus plantarum* initially increased and then decreased. In LS, the relative abundance of *Lactiplantibacillus plantarum* also increased and then decreased with increasing packing density, peaking at a density of 500 kg/m^3^. The relative abundance of *Lentilactobacillus* decreased with the decrease in packing density. Conversely, the relative abundance of *Lentilactobacillus* decreased as packing density decreased. However, the total relative abundance of these two genera showed a decreasing trend overall. Previous studies have indicated that the relative abundance of *Lactiplantibacillus plantarum* in sweet sorghum silage inoculated with LAB decreases with increasing packing density [23]. Additionally, because *Lentilactobacillus* has higher acid tolerance, its relative abundance tends to increase during the later stages of fermentation [27]. The total relative abundance of *Lactiplantibacillus plantarum* and *Lentilactobacillus* in CK was higher than in LS, possibly due to the lower pH resulting from additive treatment, which inhibits the growth of *lactobacilli*.

At a packing density of 500 kg/m^3^, the relative abundance of *Lactiplantibacillus plantarum* and *Lentilactobacillus* in the additive treatment group was significantly higher than in the CK group, suggesting that these bacteria may work synergistically at this density to promote a more efficient fermentation process. However, this study has some limitations. First, although the additive showed positive effects on microbial diversity and fermentation quality, its specific mechanisms of action require further investigation. Second, this study only assessed the effects of a specific combination of additives, and future research could explore the impact of other additives or combinations on rape straw silage. These findings have important implications for animal husbandry. By optimizing the silage process, we can not only enhance the feed value of rape straw but also reduce agricultural waste and promote sustainable agricultural development. Moreover, this study provides valuable insights that could be applied to the silage of other crop residues.

## 5. Conclusions

Increasing the packing density and using LAB inoculants significantly improved the fermentation quality of rape straw silage. The optimal packing density was observed at 500 kg/m^3^ FW, which effectively reduced fermentation losses and enhanced the relative abundance of beneficial bacteria, such as *Lactiplantibacillus plantarum* and *Lentilactobacillus*, while suppressing undesirable bacteria like *Enterobacteria*. These results suggest that applying a packing density of 500 kg/m^3^, along with LAB additives, can optimize silage fermentation, enhance nutrient preservation, and provide a practical solution for improving the quality and sustainability of rape straw as livestock feed.

## Figures and Tables

**Table 1:** Effect of additives and packing density on fermentation weight loss during the fermentation process (*n* = 3).

Items	Ensiling Days (g/kg FW)					SEM	*p*-Value
	1 d	3 d	6 d	20 d	45 d		
CK 450	0.104 bcE	0.376 cD	0.576 bcC	1.13 bB	1.80 bA	0.162	<0.001
LS 450	0.201 aD	0.722 aC	0.926 aC	1.53 aB	2.43 aA	0.207	<0.001
CK 500	0.112 bcE	0.388 cD	0.595 bcC	1.14 bB	1.82 bA	0.163	<0.001
LS 500	0.159 abE	0.486 bD	0.633 bC	1.07 bB	1.69 bcA	0.142	<0.001
CK 550	0.080 cE	0.352 cD	0.524 cC	1.01 bcB	1.66 bcA	0.149	<0.001
LS 550	0.100 cD	0.402 cC	0.527 cC	0.918 cB	1.40 cA	0.122	<0.001
SEM	0.012	0.031	0.035	0.050	0.082		
*p*-value	0.003	<0.001	<0.001	<0.001	<0.001		
Internation	D	L	T	DxL	DxT	LxT	DxLxT
*p*-value	<0.001	<0.001	<0.001	<0.001	<0.001	<0.001	<0.001

Note: CK, rape straw ensiled at 450, 500, and 550 kg/m^3^ (CK 450, CK 500, CK 550); LS, rape straw ensiled with LAB and sugar at the same densities (LS 450, LS 500, LS 550). Different uppercase letters (A, B, C, D, E) indicate significant differences among ensiling days within the same treatment (*p* < 0.05). Different lowercase letters (a, b, c) indicate significant differences among treatments on the same day (*p* < 0.05). D, silo density; L, additives; T, ensiling time.

**Table 2:** Effect of additives and packing density on pH value and organic acid concentrations in rape straw silages (*n* = 3).

	pH	LA (g/kg DM)	AA (g/kg DM)	LA/AA	PA (g/kg DM)	BA (g/kg DM)	AN (g/kg)
CK 450	6.24 a	4.34 c	15.4	0.279 b	2.76 a	2.27 b	0.736 a
LS 450	4.95 b	30.7 b	11.0	2.92 a	ND b	ND c	0.338 c
CK 500	6.30 a	2.01 c	12.2	0.165 b	1.63 ab	2.42 b	0.625 b
LS 500	4.90 bc	38.3 ab	11.1	3.76 a	ND b	ND c	0.322 c
CK 550	6.25 a	ND c	12.7	ND b	1.41 ab	5.38 a	0.633 b
LS 550	4.75 c	46.3 a	12.2	4.09 a	0.643 b	ND c	0.355 c
SEM	0.171	4.67	0.623	0.475	0.292	0.500	0.042
*p*-value	<0.001	<0.001	0.384	0.001	0.013	<0.001	<0.001
D	0.124	0.245	0.599	0.747	0.533	0.008	0.094
L	<0.001	<0.001	0.130	<0.001	0.001	<0.001	<0.001
DxL	0.158	0.026	0.384	0.504	0.172	0.008	0.101

Note: CK, rape straw ensiled at 450, 500, and 550 kg/m^3^ (CK 450, CK 500, CK 550); LS, rape straw ensiled with LAB and sugar at the same densities (LS 450, LS 500, LS 550). LA, lactic acid; AA, acetic acid; LA/AA, lactic acid to acetic acid ratio; PA, propionic acid; BA, butyric acid; AN, ammonia nitrogen; Different lowercase letters (a, b, c) indicate significant differences among treatments (*p* < 0.05). D, silo density; L, additives; DxL, interactions between packing density and inoculation.

**Table 3:** Effect of additives and packing density on microbial counts in rape straw silages (*n* = 3).

	LAB (lg cfu/g FW)	Yeasts (lg cfu/g FW)	Coliforms (lg cfu/g FW)	Bacteria (lg cfu/g FW)
CK 450	9.11	8.75	5.15 a	8.78 a
LS 450	8.99	8.34	4.31 b	8.01 a
CK 500	9.00	8.64	5.53 a	8.69 a
LS 500	9.11	8.39	<2.00 c	6.62 b
CK 550	9.10	8.63	4.99 ab	8.69 a
LS 550	9.04	7.31	<2.00 c	6.21 b
SEM	0.039	0.194	0.584	0.294
*p*-value	0.932	0.293	0.107	0.003
D	0.983	0.382	<0.001	0.121
L	0.801	0.097	<0.001	<0.001
DxL	0.577	0.458	<0.001	0.176

Note: CK, rape straw ensiled at 450, 500, and 550 kg/m^3^ (CK 450, CK 500, CK 550); LS, rape straw ensiled with LAB and sugar at the same densities (LS 450, LS 500, LS 550). Different lowercase letters (a, b, c) indicate significant differences among ensiling days within the same treatment (*p* < 0.05). Different lowercase letters (a, b, c) indicate significant differences among treatments (*p* < 0.05). D, silo density; L, additives; DxL, interactions between packing density and inoculation.

**Table 4:** Effect of additives and packing density on dry matter content and nutritional compositions concentration in rape straw silages (*n* = 3).

	DM (g/kg)	WSC (g/kg DM)	CP (g/kg DM)
CK 450	394 b	0.560 c	38.6 bc
LS 450	419 a	1.12 b	45.2 a
CK 500	398 b	0.84 bc	37.6 bc
LS 500	411 a	0.89 bc	43.1 a
CK 550	380 c	0.85 b	37.3 c
LS 550	414 a	1.33 a	40.3 b
SEM	3.47	0.122	0.077
*p*-value	<0.001	<0.001	<0.001
D	0.040	<0.001	0.013
L	<0.001	0.00139	<0.001
DxL	0.032	0.012	0.160

Note: CK, rape straw ensiled at 450, 500, and 550 kg/m^3^ (CK 450, CK 500, CK 550); LS, rape straw ensiled with LAB and sugar at the same densities (LS 450, LS 500, LS 550). Different lowercase letters (a, b, c) indicate significant differences among ensiling days within the same treatment (*p* < 0.05). Different lowercase letters (a, b, c) indicate significant differences among treatments (*p* < 0.05). D, silo density; L, additives; DxL, interactions between packing density and inoculation.

**Table 5:** Effect of additives and packing density on sequencing data and alpha diversity of bacteria in rape straw silages (*n* = 3).

	Raw Tags	Valid Tags	Observed Otus	Shannon	Simpson	Chao1	Goods Coverage	Pielou_e
freach	84,063	65,144 b	334 a	5.46 a	0.943 a	338 a	1	0.657 a
CK 450	84,289	69,917 ab	160 b	4.20 b	0.880 ab	160 b	1	0.587 ab
LS 450	85,638	73,589 a	196 ab	3.44 bcd	0.823 b	199 ab	1	0.453 cd
CK 500	82,711	69,278 ab	171 b	4.23 b	0.893 a	172 ab	1	0.580 ab
LS 500	84,074	74,119 a	206 ab	2.61 d	0.683 bc	209 ab	1	0.343 e
CK 550	83,181	68,542 ab	205 ab	3.80 bc	0.803 b	205 ab	1	0.510 bc
LS 550	82,406	74,915 a	184 ab	3.05 cd	0.773 bc	188 ab	1	0.407 de
SEM	490.6	984.6	19.5	0.212	0.021	19.8 ab	—	0.024
*p*-value	0.696	0.044	0.262	<0.001	0.004	0.271ab	—	<0.001
D	0.330	1.00	0.937	0.333	0.218	0.938	—	0.083
L	0.592	0.018	0.654	0.001	0.009	0.621	—	<0.001
DxL	0.701	0.831	0.780	0.287	0.084	0.790	—	0.080

Note: CK, ensiled at 450, 500, and 550 kg/m^3^ (CK 450, CK 500, CK 550); LS, rape straw ensiled with LAB and sugar at the same densities (LS 450, LS 500, LS 550). Values with different lowercase letters (a, b, c, d and e) indicate significant differences among treatments (*p* < 0.05); D, silo density; L, additives; DxL, interactions between packing density and inoculation.

## Data Availability

Data are contained within the article.
